# Chicago Society of Physicians and Surgeons and Government Report on Cholera*Published in accordance with the resolution of the Society.

**Published:** 1876-05

**Authors:** I. N. Danforth, J. N. Hyde

**Affiliations:** Chicago; Chicago


### THE CHICAGO SOCIETY OF PHYSICIANS AND SURGEONS AND THE GOVERNMENT REPORT ON CHOLERA.*

BY I. N. DANFORTH, M.D., and J. N. HYDE, M.D., OF CHICAGO.

We desire to call the attention of the medical profession to the volume entitled, "The Cholera Epidemic of 1875 in the United States," which was recently issued from the Government Press. It was prepared in accordance with an Act of Congress, passed on the 25th of March, 1874, and is, in fact, a compilation of Reports from the Secretaries of the Treasury and the War Departments, transmitted to the Senate and House of Representatives by the President of the United States in a message bearing date January 13, 1875.

* Published in accordance with the resolution of the Society.

We do not propose to review this work, as that labor has been carefully and conscientiously performed by numerous critics connected with the medical press of our country. We desire merely to comment upon the fol lowing statement to be found on the 37th page of the work :

"Through the kindness of Dr. Ben. C. Miller, Sanitary Superintendent of Chicago, we are able to present the results of a series of microscopic examinations which were made at his request by Dr. I. N. Danforth, of Chicago, the autopsies from which the specimens examined were obtained having been made by Dr. Marshall W. Wood, now of the U. S. Army. These reports are possessed of an additional interest from the fact that they are as yet the only published American investigations conducted in the epidemic of 1873."

During the summer of the year named, an epidemic visited a limited district of the city of Chicago lying just beyond its southern limits. It was soon after announced in an open meeting of the Chicago Society of Physicians and Surgeons, that cholera had made its appearance in the neighborhood of 37th and Burnside (now South Dearborn) streets. A resolution was promptly passed, requesting all members practicing in that locality, to report their experience in full. The response was equally prompt and full of interest. Minute details were given, respecting the origin and extension of the disease, together with the history of several typical cases. Dr. W. M. Boyd at one meeting exhibited a map indicating not only the houses and streets of the infected locality, but also showing the line traced by the advance of the epidemic. These reports awakened great interest among the members of the Society, who attended all its meetings in large numbers. One special meeting was called and held for the sole purpose of discussing the questions relative to the epidemic malady. The need of complete investigation and elaborate reports, was appreciated by all. On the 18th day of August, 1873, two committees were, by unanimous resolution, appointed for this purpose. They were constituted as follows: "On the History, Nature and Contagiousness of the Disorder, Dr. Thomas Bevan, chairman, Drs. John Bartlett, C. J. Simons, I. N. Danforth and Lester Curtis. On Prophylaxis and Treatment, Dr. J. H. Etheridge, chairman, Drs. H. P. Merriman, W. M. Boyd and F. H. Davis."*

Dr. Hyde was then secretary of the Society, and was naturally interested in forwarding, as far as possible, the work which had thus been inaugurated. On the evening of Aug. 27, a hasty and imperfect post-mortem examination had been made by a medical student, of the body of a patient, dead of cholera in the district of the city to which reference has been made. From this young and inexperienced student, Dr. M. W. Wood secured four specimens, removed from the body, and the merest scrap of information relative to the ante-mortem history of the case. These few facts and specimens were brought to Dr. Hyde as secretary of the Society, and by him transmitted to Dr. I. N. Danforth, who, according to previous arrangement, had been charged with the report on the pathology of the disease. Dr. Danforth erroneously concluded that Dr. Wood had conducted the autopsy referred to, and felt, very properly, that the city of Chicago should not permit its public institutions to be administered in so lax a manner. As the patient had died in a Cholera Hospital, he naturally supposed that the examination and report had been made by an officer of that hospital. In his report, therefore, Dr. Danforth characterized the clinical history of this case as "provokingly meagre and deficient, especially when it is taken into consideration, that the patient died in a public hospital, subject to the disciplinary regulations thereof."

We refer to these facts in order to save our friend, Dr. M. W. Wood, from the imputation of having made this defective and slovenly post-mortem examination, as it

* This is a transcript from the Records of the Chicago Society of Physicians and Surgeons.

would appear from the Government Report. Dr. M. W. Wood is known to us as a careful and conscientious surgeon, who is to-day an ornament to the medical staff of the army as he is to the membership of the medical organization that had undertaken the investigations in question.

Conscious of the need of further material, and aware of the meagre character of the results afforded by this first examination, Dr. Hyde offered to conduct a second necroscopy, should opportunity for such be offered. Accordingly, at 10 A. M., of Sept. 9, 1873, Dr. Hyde, at the request and in the presence of special sanitary inspector Dr. Chas. J. Simons and Dr. M. W. Wood, made a section of the body of Joseph Schere in the Cholera Hospital. At this time every viscus of the cranial, thoracic and abdominal cavities was carefully inspected and dissected, and a specimen of each forwarded to Dr. Danforth with eleven cholera stools, separately bottled and consecutively numbered, together with notes of the gross appearances of the organs and as much of an ante-mortem history as could be obtained, not only at the hospital but at the other places visited by the patient before death.

It is made to appear in the Government Report that Dr. Hyde merely served as a clinical clerk on this occasion, though the record of the autopsy is given in his language. Neither Dr. Wood nor Dr. Simons ever claimed to have made this autopsy. The labor incidental to its performance occupied the entire forenoon, and Dr. Hyde was for two days afterward prostrated by cholera-like discharges, which speedily yielded to proper treatment.

The question considered above, however, is of much less importance than that which follows, viz.: "*Can the Board of Health of Chicago justly claim that these examinations were conducted under their direction, and thus appropriate the results to which they led?*" This is the question which has led us to present the facts in the case to the notice of the profession.

The following extracts copied from newspaper clippings of daily journals of Chicago, published during the prevalence of the epidemic, may serve to indicate the part taken by the Board of Health at that time.

The first—a secular report of an open meeting of the Society of Physicians and Surgeons—reads as follows: "Dr. Bartlett thought the disease was *Cholera Asiatica* and nothing else. He wanted to know what the Board of Health were doing and what they were paid for, if they permitted such a state of affairs as that spoken of by Drs. Boyd and Jackson. The condition of many parts of the city was outrageous."

The "state of affairs" to which Dr. Bartlett had referred, was that which had been graphically described by medical men practicing in the infected locality, they having testified before the Society, that, in some instances, cholera dejections, neither deodorized nor disinfected, had been thrown out upon the streets, and there suffered to remain, the fruitful parents of a progeny of disease-germs. At another time, it had been stated that feather beds, upon which patients of German nationality had evacuated the contents of their bowels and subsequently died, had been, without any interference, transported from one locality to another on the removal of the survivors of the family from the infected to a more salubrious district.

Whether the Board of Health were finally aroused by the appearance of these startling statements in the public press of the city, we do not presume to decide. But it was no secret, at the time, that the Board desired medical men to report patients who were the victims of the epidemic disease, as dead of *cholera morbus*. It is somewhat doubtful whether they believed cholera to have actually affected the city. Here is a second extract, illustrative of this point:

"Dr. A. R. Jackson said it was an important point to determine whether these cases should be acknowledged as cholera, or whether, *as demanded by the Board of Health*, they should continue to call it cholera morbus."

These words were taken down by a reporter of the daily press then present.

On the appearance of these details in the public journals, one daily newspaper, which chanced not to be represented at this particular meeting, soon after published an article headed "No Asiatic Scourge!!" From it the following extract is appended:

"The sanitary superintendent w first sought, but found to be housed on account of a slight indisposition. His secretary who keeps the records and compiles the statistics of the Board of Health, said that no cholera cases had been reported from any part of the city, and that there had been no deaths from the disease."

Subjoined was a tabulated statement, evidently taken from the records of the Board, going to show that the health of the city was unprecedentedly good, and that the rate of mortality was even less than during the same season of the preceding year. If the figures did not lie, Chicago decidedly was innocent of cholera.

Very different was the sentiment entertained by a large portion of the members of the Society of Physicians and Surgeons, some of whom were practicing in the infected locality, and many of whom visited it for the purpose of confirming the facts here reported. The view, however, held by Dr. Wood at this time was essentially different from that of the others, and is well set forth in his able and interesting letter published in the Government Report.

After the epidemic had completely subsided, the sanitary superintendent, presumably for the first time, learned of the investigations pursued by the Society. He then communicated with Dr. Hyde, requesting to be allowed the use of the papers prepared on the subject for a report which he was about to make to the National Health Association in New York.

His request was cheerfully complied with. Dr. Hyde at once called upon Dr. Danforth, and, as the secretary of the Society, requested the latter to furnish the required material. Subsequently the statement was made to Dr. Danforth, by one high in authority, that the Board of Health were parties to the investigation and thus entitled to make use of its results. Dr. Danforth, thus assured, permitted his manuscript to go out of his hands.

We presume that it was incorporated into the report made by the sanitary superintendent to the Health Association in New York ; and hope that in the latter, credit was given where credit was due.

In his report, however, to the Board of Health of the city of Chicago, dated March 31, 1874, and published in book form during the same year, these reports are incorporated in full, and prefaced with the following statement:

"A microscopic examination of Cases II and III was made by Dr. Danforth *at my request*, who has kindly furnished notes and drawings of the examination."

On the evening of February 9, 1874, Dr. Danforth in due form presented to the Chicago Society of Physicians and Surgeons his written report of the sub-committee on the pathology of the epidemic as it had occurred here. His paper was, at his request, read by Dr. Bridge, and he in the meantime, exhibited his microscopical sections of the intestinal membrane, etc., illuminated by the oxy-hydrogen light and explained their various appearances as they passed beneath the objectives. The meeting was very largely attended not only by members of the Society, but also by invited guests who had assembled in consequence of the unusual interest of the proceedings. After the reading and exhibition of the specimens were concluded, the report was duly accepted, and Dr. Danforth received, by resolution, the unanimous thanks of the Society.

The introductory pages of this report are subjoined, not only as showing the circumstances under which it was prepared, but also as determining what was suppressed or elided from the manuscript which had been previously loaned to the officials of the Board of Health and by them published in a fragmentary manner.

"REPORT ON THE PATHOLOGICAL ANATOMY AND HISTOLOGY OF CHOLERA. By *I. N. Danforth, M.D.*, Lecturer on Pathology in Rush Medical College. Read before the Chicago Society of Physicians and Surgeons, Feb. 9, 1874.

Early in the summer of 1873, epidemic cholera made its appearance in the city of New Orleans, and, in spite of vigorous sanitary measures, it rapidly spread in all directions. It swept northward through the Mississippi valley, capturing and conquering city after city, and hamlet after hamlet, in defiance of the fact that civic authorities had entrenched themselves behind the most approved measures of prophylaxis, and in direct violation of its own time-honored law of migration. In the month of August it reached Chicago, and became "endemic" in the so-called Bridgeport district. Probably in no part of Chicago are the sanitary conditions so desperately bad as in the section above mentioned. As the duty of reporting at length thereupon has been assigned to another member of the committee, I may dismiss the subject by remarking that the habitual drinking of surface water for want of hydrant water, the enforced dependence upon surface drainage in default of underground sewerage, and the total absence of pavements upon the streets, form the main elements of a problem so easy of solution that it is scarcely a problem at all.

On the 18th of August, 1873, the "Chicago Society of Physicians and Surgeons" appointed a committee, whereof Dr. Thomas Bevan was chairman, to investigate the "history and nature of the disease now prevailing in the south-west district of the city, with especial reference to the question of contagion." The committee consisted of Drs. Bevan, Bartlett, Danforth, Simons and Curtis; a division of labor was subsequently made, and to the writer was assigned the intrinsic pathology and microscopy of the disease in question. The infected districts was so far from my own residence that it was impossible for me to visit it for the purpose of making autopsies. I was therefore reduced to the necessity of taking what morbid specimens I could get, and make my report thereupon. Through the kindness of Dr. James Nevins Hyde, I have been furnished with the histories of two cases, with specimens of evacuations (both ante- and post-mortem), and several specimens of intestine and other organs. In the following report, I have entered upon no speculations, but have endeavored to faithfully and somewhat minutely record the gross and microscopic appearances, with the hope of adding somewhat to our knowledge, particularly in the domain of pathological histology."

With reference to Case II, it is said :

"Dr. Hyde * * * furnished me the clinical history so far as he could obtain it, and very complete notes of a very complete and workmanlike post-mortem examination made by himself. I cannot do better than insert Dr. Hyde's account of the case at length, which is as follows : "

When Dr. Ely McClellan of the U. S. Army visited Chicago during the period in which he was engaged in the compilation of his part of the "Report on Cholera," issued by the Government, he was a guest of the sanitary superintendent. At the request of the requisite number of the members of the Chicago Society of Physicians and Surgeons, Dr. Hyde, who was then also the secretary of the Society, called the special meeting at which Dr. McClellan presented his highly interesting sketch of the progress of the disease throughout the State of Kentucky. This meeting was also largely attended, and the speaker took occasion to pay a deserved compliment to the Society by stating that he had collected no reports which equaled, in interest and value, those obtained from the investigations conducted under its auspices.

Our purpose is now evident. It is to call the attention of the profession to the fact that the part taken by the Society of Physicians and Surgeons is coolly and completely ignored in a volume of 1025 pages published under the auspices of the Government and in accordance with a joint resolution of both Houses of Congress. Or, rather, not being ignored, the valuable portion of its report is as coolly and completely appropriated and credited to the direction of the Chicago Board of Health.

If any deem that this subject is too trifling to require the detailed explanation and correction we have here endeavored to make, we beg leave to recall to such the comment of the "Report" itself, as published by the Government, upon the researches in question, which are pronounced to be "*possessed of an additional interest from the fact that they are as yet the only published American investigations conducted in the Epidemic of* 1873."

We desire to be understood as charging no one with a breach of good faith, common honesty or professional honor. We desire, in fact, to make no charges whatever. We simply claim to set forth the facts in the case which are briefly these: 1st, That the Board of Health of Chicago had not in any way the slightest connection with the work of inaugurating or prosecuting these researches; and, 2nd, That the latter were undertaken under, and solely under, the auspices and direction of the Chicago Society of Physicians and Surgeons by the instrumentality of its duly appointed committees. To it, therefore, and to no other body, belongs the honor, if any there be, of the work accomplished. Had not these committees been appointed, it may well be doubted whether the investigations would have ever been set on foot; certainly the work performed respectively by the authors of this statement was not undertaken as private individuals, but as members of the Society. The specimens examined were furnished to Dr. Danforth with that specific understanding, and were examined and reported upon accordingly.

One other correction remains to be made. The formula for the prophylaxis of cholera which was successfully used in the infected district and which is published in the Government Report, was furnished by Dr. Thomas Bevan, and formed part of the report of the committee on Prophylaxis and Treatment, which was also made in due form to the Society of Physicians and Surgeons.

We have thus presented the statement given above, in a desire to do impartial justice to all concerned, and to make a correct historical record of the facts in the case.

